# Homozygous missense variant F12 (Gly506Asp) associated with severe factor XII deficiency: a case report

**DOI:** 10.1186/s13256-023-04238-9

**Published:** 2023-12-07

**Authors:** Mansour Aljabry, Aljoud Algazlan, Nouf Alsubaie, Shatha Bin Dher, Hassan Semar Aljabri, Ghazi S. Alotaibi

**Affiliations:** 1 Department of Pathology, College of Medicine, King Saud University, P.O Box2925, Riyadh, 11461 Kingdom of Saudi Arabia; 2 Premarital Examination Center, Madina Region, Ministry of Health, Al-Madinah Al-Munawwarah, Saudi Arabia; 3 Division of Hematology/Oncology, Department of Medicine, College of Medicine, King Saud University, Riyadh, Saudi Arabia

**Keywords:** Rare factors, FXII, Molecular

## Abstract

**Background:**

Factor XII deficiency can be related to either homozygous or compound heterozygous pathogenic variants in the F12 gene. The disease is commonly known as Hageman trait and is inherited in both autosomal recessive or dominant patterns. Clinically, factor XII deficiency is not associated with bleeding but conversely has been linked to thrombotic events, recurrent pregnancy loss, and hereditary angioedema. Molecular data of F12 deficiency are scarce and have revealed varying results between cases. However, most of the reported variants are missense mutations, gross deletions, or small insertion. Factor XII deficiency has been reported in the Saudi population in several studies, either as isolated case reports or included within the studies of rare bleeding factors deficiency. However, molecular data are lacking as no case report of genetic studies related to factor XII deficiency has been published in our local population, to the best of our knowledge.

**Case report:**

Herein we describe a homozygous missense variant involving exon 12 within F12 gene (5:176,830,269 G>A; p.Gly506Asp) in a 36-year-old Saudi multiparous female referred from the surgical clinic with significantly high activated partial thromboplastin time during preoperative assessment for sleeve gastrectomy. The patient had no history of bleeding episodes during the previous deliveries nor any tooth extractions. She had single event of spontaneous abortion during the 15th week of gestation without any bleeding complication. There was no history of thrombosis or skin manifestations, and she was not taking any medicines. There was no family history of bleeding or thrombosis. Family history revealed consanguinity as the parents are first-degree cousins. Physical examination was unremarkable. Upon investigation, the prolonged activated partial thromboplastin time was fully corrected by a 1:1 mixing study with normal pool plasma while lupus anticoagulant tests were negative. Factor assays and von Willebrand factor tests are all within normal ranges except for factor XII, which was severely deficient. A homozygous missense variant involving exon 12 within F12 gene (5:176,830,269 G>A; p.Gly506Asp) was identified.

**Conclusion:**

F12 (5:176,830,269 G>A; p.Gly506Asp) variant is likely to be a pathogenic variant among homozygous factor XII-deficient patients. Genetic counseling and management of the patients and families should be based on clinical evaluation.

## Introduction

Factor XIIa plays a key role in converting prekallikrein to kallikrein, which in turn cleaves factor XII (FXII) to heavy chain alpha-factor XIIa and light chain beta-factor XIIa. Alpha-factor XII helps in initiation of fibrinolysis system by converting plasminogen into plasmin, resulting in fibrin degradation. Furthermore, FXII has an integral role in generation of bradykinin, a protein that promotes inflammation by increasing vessel wall permeability [1–3].

The FXII gene is 12 kb with 13 introns and 14 exons and mapped to chromosome 5q35.3. Homozygous or compound heterozygous pathogenic variants in the F12 gene cause factor XII deficiency giving rise to Hageman trait, which is more often a rare autosomal recessive trait that is usually an incidental finding during preoperative assessment of isolated prolonged activated partial thromboplastin time (aPTT) [4, 5].

According to the level of FXII antigen, this deficiency is divided into three categories: a cross-reacting material (CRM)-negative group (undetectable FXII:Ag), a CRM-positive group (normal FXII:Ag), and a CRM-reduced group (decreased FXII:Ag) [6]. Clinically, factor XII deficiency is not associated with bleeding but conversely has been linked to thrombotic events, recurrent pregnancy loss, and hereditary angioedema [7].

Genetic analysis of different cases has revealed varying results. Majority of mutations are missense/nonsense, followed by small deletions, regulatory splicing, small insertions, and gross deletions [3, 4].

Factor XII deficiency has been reported in the Saudi population in several studies either as isolated case reports or included within the studies of rare factor deficiency [8]. However, molecular data are lacking as no case report of genetic studies related to FXII deficiency have been published, to the best of our knowledge^.^

In the present study, we describe a multiparous female patient with a severe factor XII deficiency and an isolated event of spontaneous abortion without significant bleeding or thrombotic complications. A homozygous missense mutation (5:176,830,269 G>A; p.Gly506Asp) was identified.

## Case report

A 36-year-old Saudi multiparous woman (gravda 4 para 3) was referred from the surgical clinic with significantly high aPTT during preoperative assessment for sleeve gastrectomy. The patient had no history of bleeding episodes during the previous deliveries or tooth extractions. She had single event of spontaneous abortion during the 15th week of gestation without any bleeding complication. There was no history of thrombosis or skin manifestations, and she was not taking any medicines. There was no family history of bleeding or thrombosis. Family history revealed consanguinity as the parents are first-degree cousins. Physical examination was unremarkable.

On a complete blood count (CBC), investigation revealed: white blood cell (WBC) 5.2 × 10^9^/L, hemoglobin 12.2 g/dL, and platelets 293 × 10^9^/L. Coagulation profile revealed: prothrombin time 13.7 seconds. (12.10–15.70), INR 1.0 (0.8–1.2), and thrombin time 15 seconds (12–19), while her aPTT was > 180 seconds (25.7–39.50). The prolonged aPTT was fully corrected by 1:1 mixing study with normal pool plasma. Lupus anticoagulant (LA) tests including PTT-LA, Staclot-LA and DRVVT were negative. Factor assays and von Willebrand factor (vWF) tests were all within normal ranges except for FXII, which was severely deficient (Table 1).

**Table 1:** Factor assays and vWF antigen and activity

Test	Results	Normal range
Fibrinogen	3.79	2.00–4.00 gm/L
Factor VIII	127.00	60.00–150.00%
Factor IX	98.00	50.00–150.00%
Factor X	73.00	40.00–120.00%
Factor XI	107.00	75.00–155.00%
Factor XII	< 1.00 (L)	70.00–145.00%
Factor XIII	117.0	60.0–150.0%
vWF Activity	61.0	50.0–150.0
vWF Assay Ag	83.0	50.0–160.0

## Methodology for molecular study

The Blueprint Genetics F12 single gene test (version 3, 07 Oct 2019) plus sequence analysis and copy number variation analysis were performed on fresh peripheral blood sample. This test targeted protein coding exons, exon–intron boundaries (± 20 bps) and selected noncoding, deep intronic variants. This test was used to detect single nucleotide variants and small insertions deletions (INDELs) and copy number variations defined as single exon or larger deletions and duplications. A homozygous missense variant involving exon12 within F12 gene (5:176,830,269 G>A; p.Gly506Asp) was identified.

## Hospital course

The patient underwent a sleeve gastrectomy because of morbid obesity without any bleeding complications. She received prophylaxis with 40 mg of enoxaparin once daily for 7 days postoperatively. Her recovery was uneventful with no thrombotic or bleeding episodes.

## Discussion

Factor XII deficiency can be related to either homozygous or compound heterozygous pathogenic variants in the F12 gene. The disease is commonly known as Hageman trait and is inherited in both autosomal recessive or dominant patterns [9–11]. 

The current patient had a homozygous mutation involving exon 12 within the peptidase S1 domain of F12 gene; (5:176,830,269 G>A; p.Gly506Asp) is a missense point mutation, which leads to substitution of glycine to aspartate. This mutation is likely to be deleterious (i.e., disease-causing mutation), especially with presence of family history of consanguinity. However, there is currently insufficient evidence to support its disease-causing role. To the best of our knowledge, this variant has not been reported previously in correlation with FXII deficiency or in a homozygous pattern. According to the Genome Aggregation Database (gnomAD), there are 25 individuals heterozygous for the (5:176,830,269 G>A; p.Gly506Asp) variant. All the affected individuals were from south Asia and no homozygous mutation or FXII deficiency was reported [11, 12]. A missense variant in an adjacent codon, homozygous F12 c.1515G>C; p.Trp505Cys, has been reported in an individual with F12-related phenotype, suggesting that this region of the protein is functionally important [13].

Reporting genotype–phenotype correlation is recommended for future case reports of factor XII deficiency to continue to characterize this disorder. Genetic analysis of different races has shown varying results. A recent study from Russia has compared the genetic and clinical data of FXII deficiency patients with normal individuals. Six deleterious variants and 40 mutant nonpathogenic alleles were detected. The most common substitutions were F12 c.-62C>T, c.-57G>C and c.1532-1G>A with an overall frequency of 92.5% of patients with FXII deficiency in the Russian population. Moreover, three novel mutations; p.615 del C, c.1180_1181delCA, and CD218 TAT->CAT p.Tyr218His were also identified [14].

Similarly, a Chinese study examined seven members of three generations of a consanguineous family. Identical homozygous mutations of the c.1748T>A; p. Ile583Asn gene was found in five of the seven members. The original presenting case was discovered incidentally during admission due to a car accident. She developed thrombosis in the inferior vena cava and right common iliac vein during the hospital course [7].

F12 c.1681C>A; p.Gly542Ser is a pathogenic variant that was reported sporadically from Taiwan and China in unrelated patients with variable reduction of FXII levels without thrombotic or bleeding manifestations [3, 4, 13].

Although the diagnosis of FXII deficiency is typically an incidental finding during investigation of prolonged aPTT in asymptomatic patients, some cases were detected during thrombophilia screening, recurrent miscarriages, or primary abortion work-up [11, 15–17]. Moreover, factor XII deficiency was found in 20% of patients with hereditary angioedema (HAE) type III, which is a rare genetic disorder characterized by recurrent episodes of abdominal pain along with swelling of the face, hands, and feet [16]. 

## Conclusion

The 5:176,830,269 G>A; p.Gly506Asp variant is likely to be a pathogenic variant among homozygous patients. Family member testing could assist in the further classification of the variant. Genetic counseling and management of the patients and families should be based on clinical evaluation.

## Data Availability

Not applicable.
